# PD-L1-Positive High-Grade Triple-Negative Breast Cancer Patients Respond Better to Standard Neoadjuvant Treatment—A Retrospective Study of PD-L1 Expression in Relation to Different Clinicopathological Parameters

**DOI:** 10.3390/jcm11195524

**Published:** 2022-09-21

**Authors:** Olga Stanowska, Olga Kuczkiewicz-Siemion, Małgorzata Dębowska, Wojciech P. Olszewski, Agnieszka Jagiełło-Gruszfeld, Andrzej Tysarowski, Monika Prochorec-Sobieszek

**Affiliations:** 1 Department of Tumor Pathomorphology, Maria Sklodowska-Curie National Research Institute of Oncology, W. K. Roentgena 5, 02-781 Warsaw, Poland; 2 Institute of Pathology, University of Bern, Murtenstrasse 31, 3008 Bern, Switzerland; 3 Department of Computational Oncology, Maria Sklodowska-Curie National Research Institute of Oncology, W. K. Roentgena 5, 02-781 Warsaw, Poland; 4 Nalecz Institute of Biocybernetics and Biomedical Engineering, Polish Academy of Sciences, Księcia Trojdena 4, 02-109 Warsaw, Poland; 5 Department of Breast Tumors and Reconstruction Surgery, Maria Sklodowska-Curie National Research Institute of Oncology, W. K. Roentgena 5, 02-781 Warsaw, Poland; 6 Department of Translational and Molecular Oncology, Maria Sklodowska-National Research Institute of Oncology, W. K. Roentgena 5, 02-781 Warsaw, Poland

**Keywords:** breast cancer, triple negative, TNBC, PD-L1, predictive, BRCA, chemotherapy

## Abstract

Triple negative breast cancer (TNBC) is typically a high-grade breast cancer with poorest clinical outcome despite available treatment modalities with chemo-, immuno- and radiotherapy. The status of tumor-infiltrating lymphocytes (TILs) is a prognostic factor closely related to programmed death ligand 1 (PD-L1) expressed on T lymphocytes modulating antitumor immunity. Immune-checkpoint inhibitors (ICI) are showing promising results in a subset of breast cancer patients in both neo- and adjuvant settings. Pathologic complete response (pCR) after neoadjuvant treatment was found to be associated with better prognosis. We analyzed the prognostic and predictive significance of PD-L1 (SP142 assay) immunohistochemical expression on TNBC patients’ samples as illustrated by pCR with regard to its relation to treatment regimen, stage, BRCA mutational status and outcome. Furthermore, we analyzed a few other clinicopathological parameters such as age, TILs and proliferation index. The study highlighted a positive role of PD-L1 evaluation for personalized pCR probability assessment. Although considerable research was made on comparison of PD-L1 level in TNBC with different patient parameters, to our best knowledge, the relation of PD-L1 status to pCR while taking treatment regimen and stage into consideration was so far not investigated.

## 1. Introduction

Triple-negative breast cancer (TNBC) defines absence of estrogen and progesterone receptors as well as the lack of overexpression of human epidermal growth factor receptor 2 (HER2). It constitutes around 15% of breast cancers, the majority being high-grade with the poorest prognosis, not responding to targeted and endocrine therapies [1]. Chemotherapy is still the standard treatment regimen, both in neoadjuvant and adjuvant settings. Unfortunately, the outcomes remain poor. Early-stage TNBC demonstrates high risk of recurrence and advanced stage presents median survival of approximately 18 months [2]. Thus, there is still a burning need for novel approaches. Studies evaluating neoadjuvant chemotherapy in early-staged TNBCs have indicated that pathologic complete response (pCR; stage ypT0/Tis, ypN0) was associated with sustained clinical benefit [3,4]. Thus, pCR became a surrogate marker of survival.

Recently, there has been a growing interest in immunotherapy with immune checkpoint inhibitors (ICIs) emerging as a valuable treatment option. The immune checkpoint programmed death-receptor 1 (PD-1) acts as a negative regulator of T cell immune function. PD-1, expressed by T lymphocytes, interacts with programmed death-ligand 1 (PD-L1) on tumor cells, inhibiting the T cells’ proliferation and restraining their cytotoxic abilities. Inhibiting the checkpoint’s function facilitates the immune response against the neoplastic cells [5]. The frequency of PD-L1 expression among breast cancer subtypes is relatively low (10–30%), when compared to other neoplasms, e.g., non-small-cell lung cancer [6]. The expression also differs with stage or subtype of cancer. The highest PD-L1 expression demonstrates TNBC followed by the HER2-positive breast cancer. The impact of stage on PD-L1 expression is more pronounced in early cancers achieving up to 60% in early-stage TNBC [7,8,9,10]. Tumor infiltrating lymphocytes (TILs) constitute a part of the tumor microenvironment. Several studies on TILs in the neoadjuvant chemotherapy setting have demonstrated this marker’s predictive and prognostic value. Compared with other breast cancer types, high TILs are more common in TNBC. Presence of both high tumor-infiltrating lymphocytes (TILs) and programmed cell death-ligand 1 (PD-L1) expression rates in TNBC microenvironment makes immunotherapy a promising alternative/supplement for chemo-radiotherapy [11,12,13,14].

TNBC tumors are associated with pathogenic variants in the BRCA1 breast cancer susceptibility gene, with 7–20% of diagnosed patients harboring pathogenic germline or somatic variants. The association between TILs and BRCAness is also well established [15]. More recently, a trend for higher PD-L1 levels was found in tumors with BRCA1 inactivating genetic variants [16]

While immunology is gaining an important role in the treatment strategy of breast cancer, the impact of biomarkers of response to immune checkpoint inhibitors (ICI) on prognosis is still not well established. Parvathareddy et al. study revealed that PD-L1 expression is correlated with worse clinical and pathological features such as younger age, higher grade and TNBC subtype [17]. Meanwhile, high PD-L1 expression has a positive impact on overall survival, disease free survival and pCR in the TNBC subtype according to single research [5,18,19,20] However, other studies, including meta-analysis, questioned this relationship giving unclear view on prognostic value of PD-L1 [21].

Not only prognostic but also the predictive significance of PD-L1 remains elusive. Its role in prediction of good response varies between early and advanced TNBC and according to individual immune function and/or disease setting. Not all clinical trials support PD-L1 as a predictor of the efficacy of ICIs: the KEYNOTE-522 and IMpassion 031 studies in early TNBC patients showed that ICIs combined with chemotherapy had a higher pCR than placebo combined with chemotherapy irrespective of PD-L1 expression. In the KEYNOTE-522 trial, the percentages of patients with pCR in the PD-L1–positive population were around 15% higher compared to the PD-L1–negative population both among those with or without ICI in their neoadjuvant chemotherapy regimen. However, in the KEYNOTE-355 clinical trial, it was observed that as tumor PD-L1 expression increased, the therapeutic effect of pembrolizumab also rise. Furthermore, the duration of response to pembrolizumab was also prolonged with increased tumor PD-L1 expression, suggesting that the clinical benefit of pembrolizumab may be related to PD-L1 expression [3,22,23].

Atezolizumab with nab-paclitaxel was the first PD-L1 inhibitor-based regimen in TNBC approved by the European Medicines Agency (EMA) and Food and Drug Administration (FDA) for locally advanced or metastatic, PD-L1-positive TNBC after promising results of IMpassion130. The indication was then voluntarily withdrawn by the manufacturer in August 2021, due to unsatisfactory results of IMpassion131. In addition, patients with early-stage TNBC (previously untreated stage II/III) may benefit from adding ICI to their neoadjuvant treatment regimen soon, as, according to recent report from I-SPY 2 trial, it increases three times their chances for pCR [24]. The presumed benefit lies in the immune microenvironment of an early tumor that manifests, i.a., through higher frequency of PD-L1 positivity than in the metastases. This may enhance the anti-tumor action of immunotherapy.

Presently, the only FDA approved ICI is pembrolizumab. The admission was granted for combined therapy with chemotherapeutic to administer in patients with locally recurrent unresectable TNBC regardless of PD-L1 expression (KEYNOTE-522), for metastatic, PD-L1-positive TNBC (KEYNOTE-355) and as a neoadjuvant treatment for early high-risk TNBC, with continued pembrolizumab monotherapy as adjuvant therapy after surgery [3,22]

Presented study focuses on the impact of PD-L1 (SP142 assay) immunohistochemical expression on pCR and outcome among high-grade TNBC patients in relation to the clinical stage, neoadjuvant treatment regimen, BRCA mutational status, TILs, proliferation index and age.

## 2. Materials and Methods

### 2.1. Study Design

The study was approved by the Institutional Bioethics Committee (decision number: 44/2020, 20 August 2020). A retrospective review of the in-house registry was conducted to search for recent 100 core biopsy samples of newly diagnosed, pretreated TNBC in the period from 2017 to 2019. Finally, only high-grade cancers were included into the analysis, consisting of 93 core biopsy samples. Clinical features and outcomes, e.g., age, stage, treatment regimen, follow up period between 24 to 60 months, recurrence, progression, were recorded.

Stage was evaluated according to the eighth edition of American Joint Committee on Cancer (AJCC) triple-negative breast carcinoma staging system [25].

For neoadjuvant treatment, AC-Taxol protocol was used as a standard regimen for TNBC patients at that time with 4 courses of doxorubicin hydrochloride (Adriamycin) and cyclophosphamide, followed by 12 courses of Paclitaxel. Carboplatin was added to Paclitaxel in BRCA-positive patients under 60 years of age and dose-dense Adriamycin and cyclophosphamide (ddAC) received young patients with no comorbidities.

All slides were reviewed by two pathologists (OS and WO) to collect the following pathologic parameters: histological grade, TILs percentage and MIB-1 index.

TILs were counted according to the international consensus scoring recommendations [26].

### 2.2. Immunohistochemical Analysis

For immunohistochemical analysis of PD-L1 status, the recommended staining protocol and required staining procedure conveyed by Roche and available online [27] was followed. First, 3–6-μm-thick sections freshly cut from stored paraffin blocks were mounted on silane adhesive slides (Super Frost cat no 05571603001) for PD-L1 staining and negative controls. Next, sections were processed on a BenchMark ULTRA (Roche Diagnostics), deparaffinized and conditioned for 60 min at 58–60 °C. For immunostaining, we used “Ready to Use” reagents by Roche Diagnostics (Basel, Switzerland): Rabbit Monoclonal Anti-PD-L1 clone SP142 (Cat. No. 07011571001) with dedicated OptiView Detection Kit (Cat. No. 06396500001) and Negative Control Rabbit Ig (Cat. No. 05266238001).

PD-L1 expression score was evaluated on immune cells (IC). Cases with PD-L1 expression on IC occupying at least 1% of the total tumor area (including the associated intratumoral stroma and immediate contiguous peritumoral stroma) were considered positive. This scoring system was used in the IMpassion130 Study for atezolizumab in metastatic TNBC [28]. Proliferation index of tumor cells was counted on archived routinely processed and stained slides by Ki-67 antigen (clone MIB-1) (Dako Denmark A/S, Glostrup, Denmark, ready to use) from formalin-fixed paraffin embedded (FFPE) core biopsy samples.

### 2.3. Molecular Analysis

For molecular analysis of *BRCA1/2* genes mutational status, DNA was isolated from patients’ whole blood samples placed in EDTA tubes (more precisely from peripheral blood lymphocytes) using QiaAmp DNA Mini Kit (Qiagen, Hilden, Germany). *BRCA1/2* genes were analyzed using panel next generation sequencing (library preparation: Oncomine™ BRCA Research Assay Chef-Ready Library Preparation, sequencing on Ion S5 using Ion 520 Chip/Ion 520 Chef Kit, Thermo Fisher Scientific, Waltham, MA, USA). The Oncomine™ Panel enables analysis of all coding of *BRCA1/2* sequences (exon coverage depth not smaller than 500 reads) with exon-intron junctions and detection of SNVs, InDels, and large exon/gene deletions/duplications. Sequencing data analysis was performed using Torrent Suite version 5.10.1 and Ion Reporter version 5.6 (Thermo Fisher Scientific, Waltham, MA, USA). Detected variants have been named according to Locus Reference Genomic Sequence LRG_292t1 (for *BRCA1*) and LRG_293t1 (for *BRCA2*) and to HGVS guidelines (varnomen.hgvs.org (accessed on 7 August 2022)). Following databases and software were used for variant annotations and classification: GnomAD, ClinVar, HGMD Professional, Varsome, Alamut.

The control group for BRCA-mutated patients were patients tested for BRCA 1 and 2 mutation who received negative results.

### 2.4. Pathological Response to Neoadjuvant Treatment

When available, we obtained the data from post-surgical histopathologic reports, including pathological response to neoadjuvant treatment. Pathological response was assessed according to Pinder measurement guidelines and divided into three groups: (1) pathological complete response (pCR) understood as either (i) no residual carcinoma or (ii) no residual invasive tumor but ductal carcinoma in situ (DCIS) present; (2) partial response to therapy (pPR), when either (i) minimal residual disease/near total effect (e.g., <10% of tumor remaining), (ii) evidence of response to therapy but with 10–50% of tumor remaining or (iii) tumor cellularity >50% remains evident, when compared with the primary core biopsy sample, although some features of response to therapy present; and (3) no response to therapy (pNR) [29]. At the same time, pathological response was collated with residual cancer burden (RCB) as calculated from available online calculator created by MD Anderson Center faculty; RCB 0 equaled pCR, RCB I—pPR, and RCB II and III—pNR [30].

### 2.5. Statistical Analysis

Data are expressed as number (percentage) or median with interquartile range. Chi-squared or exact Fisher test was used to compare percentages, whereas differences between continuous variables were investigated using Mann-Whitney test. Relationship between two variables was analyzed by Spearman’s rank correlation coefficient (rho). Observed effect was considered statistically significant if *p*-value was lower than 0.05, unless otherwise indicated. In a multivariable analysis, the logistic regression was used to determine variables associated with pCR. Stepwise method was applied for adding or removing variables to examine dependencies between pCR and various combinations of other data. Statistical analyses were performed in R ver. 4.0.3 [R Core Team, n.d.] and in Matlab 2021a (MathWorks, Natick, MA, USA) with the ‘*stepwiseglm*’ function embedded within.

## 3. Results

### 3.1. Histological Findings

All TNBC included in the study were graded as poorly differentiated (G3) carcinomas of no special type (Table 1). The TILs score was ranging from 0% to 90% (median 25%) for all 93 cases; 33% of cases placed above the threshold of 30% (Table 1).

### 3.2. Immunohistochemical Findings

**Figure 1:**
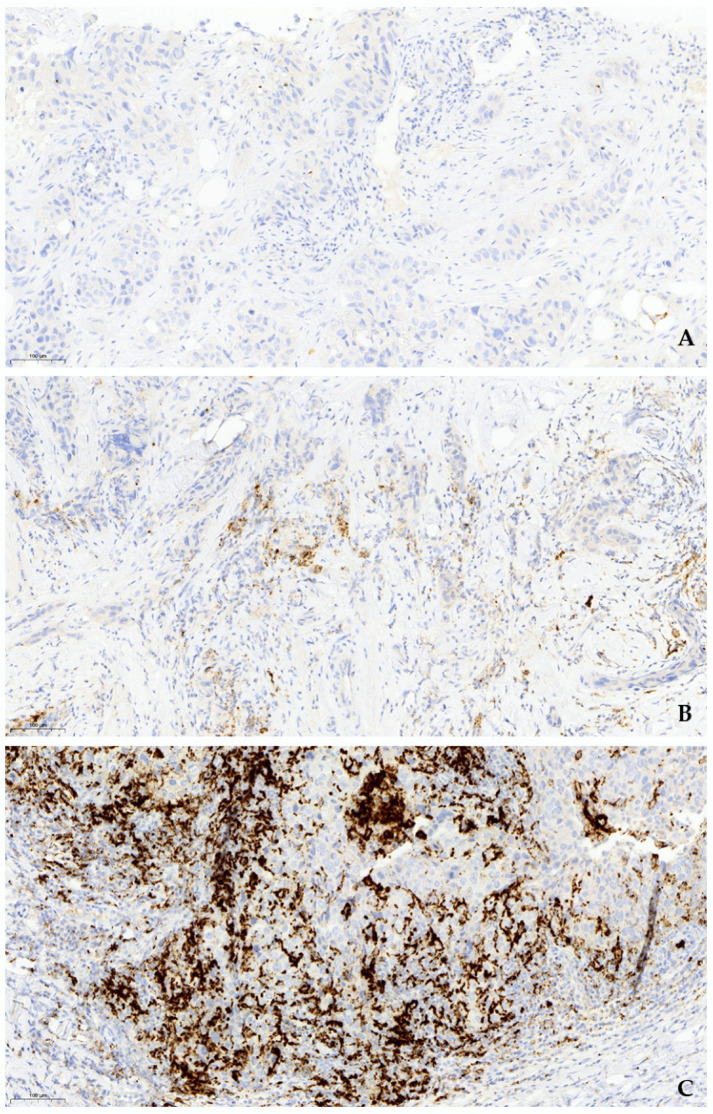
PD-L1-positivity of immune cells shows membranous and cytoplasmic staining of various intensity. A punctate or granular pattern of cytoplasmic staining is typically observed with SP142 assay (IHC PD-L1 SP142, 15× magnification). (**A**). PD-L1-negative (IC < 1%), (**B**). PD-L1- positive (IC ≥ 1%, low expression), (**C**). PD-L1-positive (IC ≥ 1%, high expression).

From 93 core biopsy samples of newly diagnosed high-risk TNBC patients, PD-L1-positivity (at least 1% expression, median 1%) (Figure 1B, C) was exhibited by 53 (57%) cases (Table 1). Obvious null PD-L1 expression was present in 65% (26/40, Figure 1A) of the negative cases, the remaining (35%) scored below 1%. One-third of positive cases scored 1% (18/53, 34%).

Median proliferation index (MIB-1) value was 75% (Table 1). Most of the cases (81.7%) surpassed the threshold of greater or even 40%.

### 3.3. Molecular Findings

BRCA 1/2 mutational status was determined in 55 cases, of which 22 were mutated.

### 3.4. Clinicopathological Findings

The age of analyzed TNBC patients ranged between 32 and 81 years (median 58.0, Table 1). Among the PD-L1 positive group, median age was 54.0, while in PD-L1 negative 61.5 years (Table 1).

Median TILs for PD-L1-positive samples were 30% compared to 15% for PD-L1-negative ones (*p* < 0.001), TILs > 30% were infrequent in the latter group (12.5%) compared to the former one (49.1%, *p* < 0.001, Table 1).

Median MIB-1 index for PD-L1-positive samples was significantly higher (80.0%) than for the PD-L1-negative group (65.0%, *p* = 0.013). MIB-1 < 40% occurred infrequently by PD-L1-positivity (7.5%) compared to the PD-L1-negativity (32.5%, *p* = 0.005).

**Figure 2:**
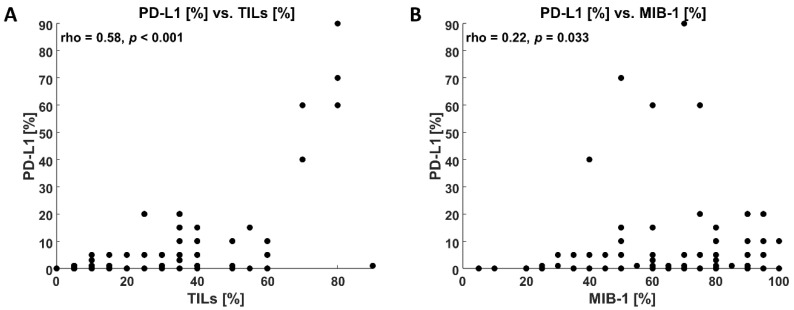
Correlations between PD-L1 and TILs (panel **A**) and PD-L1 and MIB-1 (panel **B**). Spearman correlation coefficient (rho) and *p*-value are located within the figure.

PD-L1 correlated positively with TILs (*p* < 0.001) and MIB-1 (*p* < 0.05) values (Figure 2), but TILs did not correlate with MIB-1 (*p* = 0.206). PD-L1 ≥ 1% was not related to BRCA mutational status (*p* = 0.775, Table 1).

### 3.5. Response to Neoadjuvant Treatment in Different Clinical Stage Groups with Regard to PD-L-1 Status and Chemotherapy Regimen (Standard AC-Taxol Protocol vs. Enhanced with Carboplatin or/and AC Dense-Dosed, Table 2)

**Figure 3:**
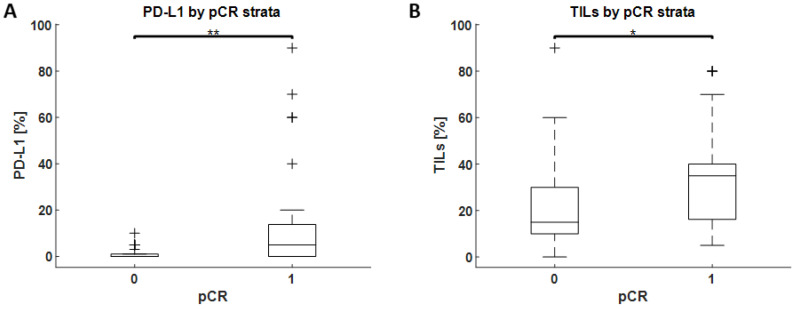
PD-L1 (panel **A**) and TILs (panel **B**) by pCR strata. ‘**’ and ‘*’ denote *p*-value < 0.01 and <0.05, respectively. On each box, the middle line indicates the median value, and the bottom and top edges of the box represent the 25th and 75th percentiles, respectively. The whiskers extend to the most extreme data points that are not considered as outliers, and the outliers are plotted individually using ‘+’ sign.

Seventy-five high-grade TNBC patients received neoadjuvant chemotherapy. The biopsies of patients with pCR presented higher percentage values of PD-L1 expression (*p*-value < 0.01) and higher TILs values (*p*-value < 0.05), when compared to partial or no response to therapy (Figure 3).

Patients *n* = 75 staged IIA-IIIC were treated with neoadjuvant chemotherapy and subsequent surgery (NChxS), 39 of them reached pCR (52%) and 9–pPR (12%). Stage IIA patients constituted 48.7% of patients with pCR. Among stage IIA patients 82.6% (19/23) achieved pCR, of whom 68% were PD-L1-positive. Of the PD-L1 negative patients who achieved pCR, 50% received enhanced treatment. The tendency was not observed in the stage IIIC group, but the cohort was also very small (20% pCR cases were PD-L1 negative).

In the IIIA-staged group, pCR was achieved by 6 PD-L1-positive and 4 PD-L1-negative patients. 100% of PD-L1-negative patients received enhanced treatment: three with carboplatin (two of them with BRCAness) and one—ddAC, in comparison to 33% of PD-L1-positive patients (two- ddAC). Of four patients staged IIIB none reached pCR and only one–pPR, who was at the same time the only PD-L1-positive patient in this group.

Among IIIC-staged patients, 80% pCR cases were PD-L1-positive, among them 50% were treated according to standard protocol, the other half constituted of BRCA-mutated patients: three were treated with addition of carboplatin and one–both carboplatin and ddAC (32 years old).

In total, 37% (15/41) of PD-L1-positive patients did not reach pCR. Among patients with pPR, 67% were PD-L1-positive. Although, in lower stages (IIA-IIIA) no significant positive impact of PD-L1-positivity could be observed, it became obvious for higher stages (IIIB-IIIC). Patients staged IIIB and IIIC that were PD-L1-negative were responding poorly to neoadjuvant treatment compared to the PD-L1-positive cohort, manifesting RCB III twice as often (Table 2). In the PD-L1 negative group with pCR, high frequency of enhanced treatment was observed (for IIIA staged patients 100% compared to 33% in the PD-L1 positive group).

**Table 2:** PD-L1 status and pathological response to treatment of cases treated with neoadjuvant chemotherapy and surgery.

	IIA		IIIA		IIIB		IIIC	
Total no of cases with nCHTH	23		24		4		24	
	PD-L1+	PD-L1-	PD-L1+	PD-L1-	PD-L1+	PD-L1-	PD-L1+	PD-L1-
pCR/RCB 0	13	6	6	4			8	2
pPR/RCB I	1	1	1	2	1		3	
RCB II		1	3	4		1	1	1
RCB III	1		2	2		2	3	6

Abbreviations: nCHTH, neoadjuvant chemotherapy; pCR, pathological complete response; pPR, pathological partial response; RCB, residual cancer burden; PD-L1, programmed death ligand 1.

### 3.6. Response to Neoadjuvant Treatment in BRCA-Mutated Patients with Regard to PD-L-1 Status and Chemotherapy Regimen (Standard AC-Taxol Protocol vs. Enhanced with Carboplatin or AC Dense-Dosed, Figure 4)

**Figure 4:**
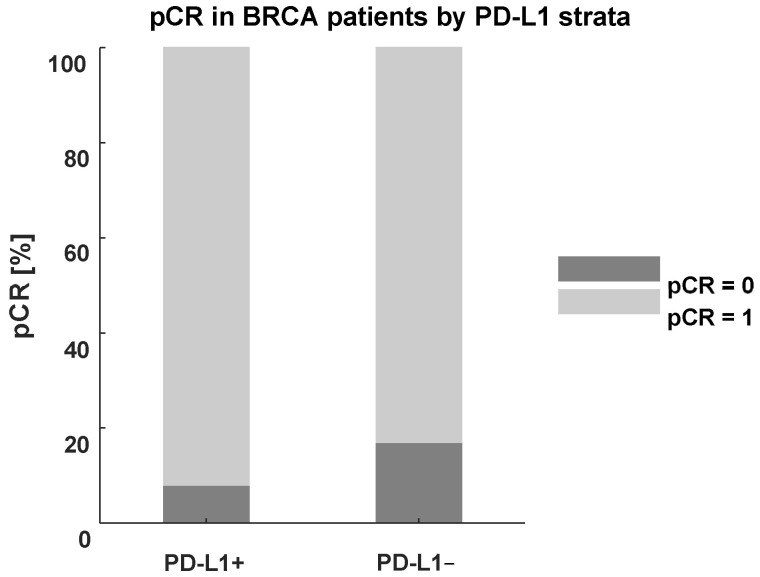
Pathological complete response (pCR) in BRCA-positive patients after neoadjuvant therapy by PD-L1 strata.

BRCAness in a TNBC-patient was not related to age, TILs, proliferation index and PD-L1 status, although median PD-L1 percentage score was slightly higher in this group. Moreover, for BRCA-mutated patients, TILs equal or greater than 30% is a good predictor of PD-L1 positivity (*p* = 0.002). Most of BRCA-mutated patients achieved pCR significantly more frequently than control (BRCA-unmutated) group (89.5% vs. 48.4%; *p* = 0.008), despite being staged IIA-IIIA only slightly more often (86.4% BRCA-mutated patients vs. 69.7% BRCA-unmutated group). The addition of carboplatin was nearly 3.5 times more frequent in BRCA-mutated compared to the control group (75.0% vs. 21.2%). However, the BRCA-mutated patients received enhanced AC doses twice less often than BRCA-unmutated subjects (15.8% vs. 35.5%). The influence of PD-L1 status on pathologic response among BRCA-mutated patients regarding their neoadjuvant treatment regimen is shown in Figure 4.

Four PD-L1-positive BRCA-mutated patients aged 32–66 (mean 56) achieved pCR despite lack of carboplatin or enhanced AC doses in their neoadjuvant therapy regimen. All these patients had tumors with TILs ≥ 30%, MIB-1 ≥ 60% and were early-stage: IIA (3) or IIIA (1). Their PD-L1 expression ranged between 5 and 15%. One of these patients was previously treated with chemotherapy (2015) for endometrial cancer.

All five PD-L1-negative BRCA-patients who achieved pCR [aged 32–69 (mean 52), staged IIA (3) and IIIA (2)] were treated with addition of carboplatin to their neoadjuvant treatment regimen. A ddAC treatment was also received by the youngest patient. Their TILs and MIB-1 ranged from <5% to 55% and 35% to 90%, respectively. One of them had a history of chemotherapy (2009) for contralateral breast cancer. Again, high frequency of enhanced treatment was observed among PD-L1 negative patients who achieved pCR compared to PD-L1 positive group.

### 3.7. PD-L1 Status and Clinical Outcome

PD-L1 status alone did not significantly affect clinical outcome. Slightly above half (52.0%) of the patients remained free of disease with the distribution between PD-L1-positive and -negative cohorts of 62.8% and 37.5%, respectively. Recurrence occurred in 13.6% of patients, equally distributed, death—in 18.3%, with slight preponderance of PD-L1-negativity (22.9% vs. 17%) and progression—in 12% of patients, also more often in the PD-L1-negative group (17.5% vs. 9.4%). Only one patient who achieved pCR/RCB 0 had unfavorable clinical outcome being at the same time a IIIC staged 60-years old with an uncommonly high PD-L1 score of 70% and TIL score of 80% after standard neoadjuvant chemotherapy with AC-Taxol protocol. Apart from this patient, only two others with RCB II progressed (both PD-L1-negative, staged IIIA and IIIC), all other women with RCB 0-II have had a favorable outcome as of June 2022 (follow up 24–60 months).

### 3.8. Multivariable Analysis

In a multivariable analysis, we searched for predictors influencing pCR. Among variables listed in Table 1 (age, stage, MIB-1 ≥ 40, 60, 80%, PD-L1 > 0%, BRCAness, TILS > 30, 40, 50 and 60%), stage and TILs > 30% were selected as the best predictors in a final model (stage III: odds ratio [OR], 0.09; 95% confidence interval [95% CI], 0.03 to 0.36, *p* < 0.001; TILs > 30%: OR, 7.47; 95% CI, 2.18 to 25.5; *p* < 0.01).

Next, we tested the addition of BRCAness to the model with the result that BRCA can affect pCR (stage III: OR, 0.17; 95% CI, 0.03 to 0.89, *p* < 0.05; BRCAness: OR, 11.17; 95% CI, 1.70 to 73.11, *p* < 0.01; TILs > 30%: OR, 6.86; 95% CI, 1.25 to 37.63, *p* < 0.05).

During subsequent testing, we removed manually TILs from the list to check what variables will be selected by an automatic stepwise regression method. Consequently, stage and PD-L1 were chosen to be associated with pCR (stage III: OR, 0.12; 95% CI, 0.03 to 0.43; *p* < 0.01; PD-L1 > 0%: OR, 2.79; 95% CI, 0.97 to 8.00; *p* = 0.05).

## 4. Discussion

The published data concerning PD-L1 positivity among TNBC and hence its predictive and prognostic significance remain inconsistent. Main reasons are different assays used (SP142, SP263, and 22C3 assays), absence of a unified scoring system [IC-tumor-infiltrating immune cells, TPS-tumor proportion score vs. CPS-combined positive score, that is the number of PD-L1 staining cells (tumor cells, lymphocytes, macrophages) divided by the total number of viable tumor cells, multiplied by 100], spatial heterogeneity of the tumor itself as well as the reliability of IHC-based detection of PD-L1 positivity [31]. PD-L1 positivity is defined per the companion diagnostic indication with any trial using a different assay and scoring system paired to a given drug. A companion diagnostic is a diagnostic test that provides required information that is essential for the safe and effective use of a therapy [32]. Most of the applied anti-PD-L1 ICIs have been approved with an associated companion diagnostics assay that was used in a decisive trial. As of August 2022, the only FDA-approved assay for TNBC remains the 22C3 Dako PharmDx IHC assay used in the KEYNOTE-086 trial. The CPS score was used as a companion diagnostic test for patient eligibility to pembrolizumab treatment. The Ventana SP142 antibody with the immune cell score IC ≥ 1% was connected to the IMPassion130 trial and atezolizumab which, although granted accelerated approval in March 2019, was then withdrawn by the manufacturer in August 2021 upon results of the IMpassion131 trial which did not meet its primary endpoint of PFS for the initial (first-line) treatment in the PD-L1-positive TNBC patient population [22]. Meanwhile, SP142 Assay is the only one approved for urothelial cancer and, together with SP263 and 22C3, for lung cancer [33]. As to scoring systems, Guo et al. made an effort to compare three of them (IC, CPS, TC— tumor cells) with the clinicopathological data using one antibody (22C3). They observed that positive PD-L1 expression by IC showed a trend for worse overall survival and distant metastasis-free survival in TNBC patients with neoadjuvant chemotherapy, which defines IC scoring system as the best prognostic marker [34].

Concerning the problem of interobserver reliability, in our study we detected lowered inter-reader reproducibility for scoring PD-L1 among gray-zone cases (slightly above and below 1%), which affected our data analysis significantly. Concerning only PD-L1 > 1%, PD-L1 was related to BRCA mutational status (*p*-value = 0.020) which was not the case for PD-L1 ≥ 1% (*p*-value = 0.775). At the same time, PD-L1-positivity stained by SP142 (IC ≥ 1%) showed less PD-L1-positive cases compared to SP263 (IC ≥ 1%) and 22C3 (CPS ≥ 1 and IC ≥ 1%) [35,36]. This result could potentially ease the interpretation of PD-L1 status by SP142 assay in a core biopsy of TNBC by accepting any robust positivity of IC as ≥1%. Of note, SP142 has been shown to have the highest concordance among readers for PD-L1 IC ≥ 1% [37].

The heterogeneity of the TNBC group is another important variable. Various studies highlight the importance of TNBC subtyping-high grade tumors in contrast to low-grade ones (mainly salivary-like carcinomas) tend to have higher rates of PD-L1 positivity due to the enhanced immunogenicity [38]. The medullary type of TNBC is well recognized to have a high PD-L1 positivity whereas the apocrine carcinoma is typically negative [39]. The material tested is also of great importance-the primary breast lesion and metastases were shown to have variable PD-L1 status. Peters et al. used SP142 antibody to evaluate breast cancer tumor blocks, when only primary, only metastatic or both primary and metastatic tumor samples were available. They received a similar PD-L1 positivity distribution, which were: 39%, 32% and 29% respectively. Among the last group tested, the discordant results occurred for primaries and metastases obtained from the same patients, which indicates the necessity of choosing the right testing sample for clinical decision-making [40]. Although Li et al. report similar prevalence of PDL1 IHC between primary and metastatic TNBC samples, their study did not compare primaries and metastases from the same patients [41].

In a multivariable analysis of our data, stage and TILs > 30% were identified as being related to pCR in logistic regression model. Omission of TILs resulted in selection of stage and PD-L1 by the regression model. Th BRCAness can be also considered as potentially associated with pCR. All three models indicate significant associations between pCR and the chosen features (all *p* < 0.001). Multivariable analysis confirms positive effect of TILs > 30%, PD-L1 and BRCAness on pCR, whereas higher stage had negative impact on pCR.

Our study is therefore concordant with the results of Wimberly et al. who showed that high expression of PD-L1 on IC was associated with a higher rate of pCR [42]. In the NeoTRIP Michelangelo randomized study, the presence of PD-L1 expression was the most significant factor influencing the rate of pCR in multivariate analysis [43]. Similarly, in the study of Oner et al. patients with PD-L1 positivity on ICs were more likely to respond to chemotherapy as measured by “MD Anderson Cancer Center Residual Cancer Burden Index” [44]. The same was confirmed in the Keynote-173 Study. In an exploratory analysis, the pCR rate showed a positive correlation with tumor PD-L1 expression and stromal TILs levels [45]. Furthermore, we observed a tendency for pCR achievement among PD-L1 positive primaries without enhanced treatment and the opposite relation for the PD-L1 negative group-here noticeably high frequency of enhanced treatment modalities were noted among PD-L1 negative patients that achieved pCR, which evoke the significance of PD-L1 status as a predictive marker of positive response to standard neoadjuvant chemotherapy-based treatment.

The impact of BRCAness on pCR can be explained by their presumed immunogenicity due to dysregulation of homologous recombination-based DNA repair, leading to increased genomic instability and higher mutational burden [46].

The role of TILs as a prognostic and predictive marker is already well established [47]. The expert panels at St Gallen 2019 and authors of the 2019 edition of the World Health Organization Classification of Tumors of the Breast recommended quantification of TILs in TNBC. The KEYNOTE-173 study showed that PDL1 CPS and stromal TILs levels were strongly correlated with each other [48]. TILs have also been associated with response to PD-1/PD-L1 inhibitors in patients with breast cancer, both pre- and on-treatment, providing real-time information that could potentially aid in guiding treatment choices [31]. TILs percentage predicts PD-L1 status. IMpassion130 exploratory analysis showed that most of the cases with TILs >20% were PD-L1-positive [45]. In our study, this was true for cases with TILs > 30%.

This study has a few limitations, being a retrospective study from a single institution with a limited number of cases. Although the majority of patients received standard neoadjuvant therapy with AC-Taxol, the remainder received enhanced treatment (carboplatin, AC dense dose), which limits the interpretation of treatment-dependent results. Furthermore, we predicted response to neoadjuvant chemotherapy without insight into the possible role of PD-L1 expression as a biomarker for predicting response to ICI as none of the patients received such treatment at the time of the study. Current protocols include carboplatin, pembrolizumab and-under 60 years of age-AC dense dose for neoadjuvant treatment of every TNBC patient [49].

More than a third of the PD-L1-positive patients (37.2%, 16/43) did not reach pCR. This group might presumably profit from adding ICI to capecitabine in the post-neoadjuvant setting. Two clinical trials are currently investigating the ICI use in adjuvant settings (NCT03756298 and NCT02954874) [50]. As of June 2022, no interim results are available for the phase 3 NSABPB-59 (NCT03281954) trial of neoadjuvant chemotherapy (paclitaxel plus carboplatin) with atezolizumab, followed by adjuvant atezolizumab and chemotherapy.

PD-1/PD-L1 interaction is only one of many factors that determines the clinical response to immunotherapeutics, as only 8–20% of metastatic PD-L1 positive TNBC patients respond to PD1/PD-L1 therapy [47]. The addition of atezolizumab to nab-paclitaxel and carboplatin did not significantly increase the rate of pCR in women with early high-risk and locally advanced TNBC as shown in the NeoTRIP Michelangelo randomized study [42] on the contrary, in the phase 3 IMpassion031 trial, by patients with early-stage TNBC, neoadjuvant treatment with atezolizumab in combination with nab-paclitaxel and anthracycline-based chemotherapy significantly improved pCR rates [51]. Furthermore, the phase 1b JAVELIN trial (NCT01772004) on avelumab reported higher response rates in PD-L1 positive versus negative TNBC patients (22.2 vs. 2.6%) using a PD-L1 cutoff of 10% [52].

A single biomarker will probably not suffice to predict clinical outcomes in response to ICI. For example, PTEN, an important regulator of DNA damage repair and mutational burden, is frequently impaired in tumors and its loss has been associated with poor response to PD-1 blockade [53]. TNBCs feature a higher tumor mutational burden and extensive genomic instability with defects in the DNA damage response [47]. However, the durable responses of a subset of PD-L1 positive patients suggest that combination treatment of immune checkpoint blockade with other treatment modalities may provide a favorable outcome.

## 5. Conclusions

Our study demonstrated that increase of PD-L1-positive IC is directly proportional to increase of TILs score and MIB-1 index. Based on our study, we set a cut-off of TILs > 30% for predicting PD-L1-positivity.

Despite the limited number of participants, this study highlighted a positive role of PD-L1 evaluation for pCR probability assessment irrespective of ICI addition to neoadjuvant therapy regimen. Particularly in the BRCA-positive group, we could observe a trend for PD-L1 status value emerging as a treatment-predictive tool. PD-L1-negative patients, having less favorable outcome understood as lower probability of pCR achievement, may require more aggressive therapy. We conclude that addition of carboplatin or enhanced AC doses in their neoadjuvant treatment regimen might have increased their chances for pCR. Contrariwise, PD-L1-positive patients more often achieved pCR with standard neoadjuvant treatment protocol. Therefore, in high-risk patients with TNBC (advanced age, comorbidities), the PD-L1-positivity might serve as a factor supporting the clinician’s decision about less aggressive therapy. Further studies on larger groups of patients are warranted to validate PD-L1 status evaluation in pathology protocols.

However, despite encouraging pCRs, progression-free and overall survival in TNBC patients is still low and the PD-L1 status did not show to influence long-term outcome.

Calculating the overall average of PD-L1 expression on IC with 1% cut-off remains a challenge for borderline cases. The result points toward possible subjectivity in decision making concerning the threshold for PD-L1-positivity in every third case in our study. Development and implementation of computational pathology methods might appropriately manage this limitation.

We encourage the systematic implementation of combined PD-L1 and TILs analyses not only for patient selection for PD-1/PD-L1 inhibition-based therapy but also as a predictive marker of response to standard neoadjuvant therapy.

## Figures and Tables

**Table 1:** Clinicopathological characteristics of patients.

Overall (*n* = 93)	PD-L1+ (*n* = 53)	PD-L1− (*n* = 40)
Age, year	54.0 (32.0–81.0)	61.5 (32.0–80.0)
Stage		
IIA (*n* = 30)	17 (32.1%)	13 (32.5%)
IIIA (*n* = 28)	16 (30.2%)	12 (30.0%)
IIIB (*n* = 4)	1 (1.9%)	3 (7.5%)
IIIC (*n* = 27)	17 (32.1%)	10 (25.0%)
IV (*n* = 4)	2 (3.8%)	2 (5.0%)
MIB-1, (%)	80.0 (25.0–100.0)	65.0 (5.0–100.0)
MIB-1 ≥ 40 (*n* = 76), no/yes (%)	4/49 (7.5/92.5)	13/27 (32.5/67.5)
BRCAness (*n* = 22), no/yes (%)	20/15 (57.1/42.9)	13/7 (65.0/35.0)
TILs, %	30.0 (5.0–90.0]	15.0 [0.0–60.0]
TILs > 30% (*n* = 42), no/yes (%)	18/35 (34.0/66.0)	33/7 (82.5/17.5)
Neoadjuvant (*n* = 75) no/yes (%)	10/43 (18.9/81.1)	8/32 (20.0/80.0)
Neoadjuvant+Carbo (*n* = 23), no/yes (%)	32/14 (69.6/30.4)	26/9 (74.3/25.7)
Neoadjuvant+ddAC (*n* = 15), no/yes (%)	32/11 (74.4/25.6)	28/4 (87.5/12.5)
pCR (*n* = 39), no/yes (%)	16/27 (37.2/62.8)	20/12 (62.5/37.5)

Abbreviations: Carbo, carboplatin; ddAC, dose-dense Adriamycin and cyclophosphamide.

## Data Availability

Clinical data are available from corresponding author on request.
